# The value of comprehensive genomic sequencing to maximize the identification of clinically actionable alterations in advanced cancer patients: a case series

**DOI:** 10.18632/oncotarget.28046

**Published:** 2021-08-31

**Authors:** Kevin Drenner, Gargi D. Basu, Laurie J. Goodman, Audrey A. Ozols, Janine R. LoBello, Thomas Royce, Michael S. Gordon, Erkut H. Borazanci, Margaux A. Steinbach, Jeffrey Trent, Sunil Sharma

**Affiliations:** ^1^Translational Genomic Research Institute (Tgen), Phoenix, AZ 85004, USA; ^2^Ashion Analytics, LLC, Phoenix, AZ 85004, USA; ^3^HonorHealth Research Institute, Scottsdale, AZ 85258, USA; ^*^These authors contributed equally to this work

**Keywords:** whole exome sequencing, RNA sequencing, precision medicine, rare mutations, genomic alteration

## Abstract

Purpose: We present seven cases of advanced cancer patients who initially underwent tumor testing utilizing smaller, panel-based tests, followed by a variety of therapeutic treatments which ultimately resulted in progression of their disease. These cases demonstrate the value of utilizing WES/RNA seq and characterization following disease progression in these patients and the determination of clinically targetable alterations as well as acquired resistance mutations.

Materials and Methods: All patients are part of an IRB approved observational study. WES and RNA sequencing were performed, using GEM ExTra^®^ on tumor and blood samples obtained during routine clinical care. To accurately determine somatic versus germline alterations the test was performed with paired normal testing from peripheral blood.

Results: The presented cases demonstrate the clinical impact of actionable findings uncovered using GEM ExTra^®^ in patients with advanced disease who failed many rounds of treatment. Unique alterations were identified resulting in newly identified potential targeted therapies, mechanisms of resistance, and variation in the genomic characterization of the primary versus the metastatic tumor.

Conclusions: Taken together our results demonstrate that GEM ExTra^®^ maximizes detection of actionable mutations, thus allowing for appropriate treatment selection for patients harboring both common and rare genomic alterations.

## INTRODUCTION

Tumor molecular profiling is increasingly standard practice for patients with advanced cancer [1]. The Cancer Genome Atlas (TCGA) and other large genetic profiling studies have identified common and rare genomic alterations, many of which have therapeutic implications [2, 3]. Alterations in the genome can predict both the therapy to which the tumor(s) may or may not respond and the aggressiveness of the disease. These components guide physicians and patients to appropriate treatment options. With the advent of comprehensive genomic testing, it stands to replace a paradigm of prescribing standard chemotherapy agents based upon the tumor of origin, histology, and stage of disease.

The field of precision medicine is continuously evolving to identify new actionable biomarkers by next generation sequencing (NGS) technologies that interrogate both DNA and RNA [4]. Clinical development studies employing precision medicine have been shown to improve overall and progression free survival as well as the tumor response rate compared with non-matched therapy [5–10]. Despite the availability of advanced technologies and an increasing understanding of the importance of genomic testing, gaps still exist in efficient patient care and access to these comprehensive tests. In a survey of Targeted Agent and Profiling Utilization Registry (TAPUR), physicians identified barriers to genomic testing which included access to tumor tissue specimens, insurance coverage, and concern that results would be non-actionable. Additional analytic aspects include long turnaround times, creating concern around timely treatment decisions, lack of education on the myriad of tests/labs to consider, and skepticism regarding test accuracy [11]. Further, studies have found that physicians may not be comfortable interpreting sequencing results or directing patient treatment based on genomic data [12, 13].

As newly available targeted treatment options become available and as clinical guidelines for actionable biomarkers are continually expanding, the need for routine use of comprehensive genomic testing for all patients has become paramount [14–17]. Therefore, panel-based tests that are considered current today may miss critical information and future actionable targets that are not included in the panel. Studies have shown that fusions and transcript variants detected by RNA transcriptome sequencing are frequently missed in tests using whole exome sequencing (WES) alone as well as in panel-based tests [18]. Cancers driven by gene fusions and translocations account for 20% of cancer mortality globally, and many of these are actionable [18]. Maximizing the detection of alterations enhances the utility of precision medicine for various solid and hematologic cancers.

We present seven patients with advanced cancers who had undergone panel-based testing and progressed on their original treatments. Upon subsequent progression, the metastatic lesions were sequenced using comprehensive WES, RNA sequencing, with tumor-normal pairing to detect both common and rare somatic alterations. GEM ExTra^®^ identified a variety of alterations resulting collectively in newly identified potential targeted therapies, mechanisms of resistance, and variation in the genomic characterization of the primary versus the metastatic tumor.

## MATERIALS AND METHODS

This observational study enrolled participants with varying stages of cancer progression. The goal of this ongoing translational research is to improve patient diagnosis, prognosis, and predictive therapy, through the study of genomic characterization of tumors by comparing different genomic testing modalities. The study, protocol # HRI-0029 is an IRB approved clinical study, WIRB # 20182804, and all patients signed informed consent to participate.

### Next generation RNA/DNA sequencing

Genomic sequencing was performed using the GEM ExTra^®^ assay (Ashion Analytics, Phoenix, Arizona, USA) in a College of American Pathologists (CAP)-accredited, Clinical Laboratory Improvement Amendments (CLIA)-certified laboratory. WES and RNA sequencing were performed on tumor samples obtained as part of routine clinical care with paired normal sequencing performed on peripheral blood to accurately identify somatic variants and to reduce false positives. Whole exome sequencing (WES) is the process of sequencing all the protein- coding regions, or exons, in a genome. WES was performed in order to identify hotspots as well as rare alterations. Details of the test methodology have been previously published [19].

The GEM ExTra^®^ test reports on somatic mutations, copy number alterations, transcript variants, and fusions. All actionable events are matched with FDA approved targeted agents as well as investigational compounds available through clinical trials. The test also detects tumor mutational burden (TMB) and microsatellite instability (MSI) to predict clinical benefit of immunotherapy [20]. The test has been validated to detect point mutations at greater than equal to 5% mutant allele frequency (MAF) (MAF greater than equal to 1% at hot spots) with an overall sensitivity of > 98%, specificity of > 99% and PPV of 100% for the detection of select single nucleotide variants (SNVs), insertion/deletion (Indels), copy number variations (CNVs), and fusions [21]. Turnaround time is within 14 calendar days from sample receipt. Clinically relevant alterations were defined as those that could be targeted using either an FDA approved drug currently on the market for any cancer type or investigational compound available through clinical trials.

## RESULTS

Of the 7 cancer patients included in this study, 2 were pancreatic cancer, 4 were Colorectal Cancer (CRC) and one patient had Gastrointestinal stromal tumor (GIST). The most frequent sources of tissue for NGS were obtained from metastatic sites. All 7 patients had undergone NGS sequencing previously and were heavily pretreated. Demographic information and details of the test findings are included in Tables 1 and 2.

**Table 1:** Patient demographics and genomic data

Case Number	Gender	Age (years)	Diagnosis	Panel Testing Result	GEM ExTra^®^ Results
1	Female	51	Metastatic malignant neoplasm of head of pancreas	biopsy #1: No Actionable Mutations biopsy #2: CREBBP (E1058fs), PBRM1 (Y417fs)	VTCN1/NRG1 fusion, TERT (c.-124C>T)
2	Female	53	Metastatic gastrointestinal stromal tissue	KIT exon 11 mutation	CDKN2A and CDKN2B Deletions, KIT (M552_K558del), KIT (N822K), KIT (Q575_Y578delinsH), KIT (Y823D)
3	Female	53	Metastatic malignant tumor of sigmoid colon	FGFR1 (H810Y), PIK3CA (F934S), TP53 (S127F)	KRAS (G12D) TP53 (S127F)
4	Female	48	Metastatic malignant neoplasm of sigmoid colon	KRAS WT	CTNNB1 (S23_D32delinsN) KRAS (A146T)
5	Female	67	Metastatic adenocarcinoma of rectal sigmoid colon	KRAS WT, EGFR-WT	APC (Q1291^*^) CDKN2A (D84N) TP53 (V143M) MET amplification
6	Male	66	Metastatic malignant tumor of colon	KRAS WT, NRAS WT, BRAF WT	APC (Q1290^*^) FGFR1 Amplification TP53 (R282W) MAP2K1 (K57T)
7	Male	54	Metastatic adenocarcinoma of pancreas	ATM (Y370fs) BRCA1 (V1804D), FGFR1 exon18 non-fusion rearrangement	ATM (Y370fs), FGFR1 amplification, FGFR1/G3BP2 fusion

**Table 2:** GEM ExTra^®^ report results

Case Number	Tumor Genomic Alterations	Clinically Actionable	
		Associated Therapies	Clinical Trials
1	VTCN1/NRG1 (fusion)	erlotinib, afatinib, pertuzumab, trastuzumab	Yes
2	CDKN2A (deletion)	abemaciclib, palbociclib, ribociclib	Yes
	CDKN2B (deletion)	None identified	Yes
	KIT (M552_K558del)	pazopanib, regorafenib, ripretinib, dasatinib, midostaurin, nilotinib, ponatinib, sorafenib	Yes
	KIT (N822K)	regorafenib, ripretinib, avapritinib, midostaurin, nilotinib, ponatinib, sorafenib Predicted non-beneficial drugs: imatinib, sunitinib	Yes
	KIT (Q575_Y578delinsH)	pazopanib, regorafenib, ripretinib; dasatinib, midostaurin, nilotinib, ponatinib, sorafenib	Yes
	KIT (Y823D)	regorafenib, ripretinib avapritinib, nilotinib, ponatinib, sorafenib Predicted non-beneficial drugs: imatinib, sunitinib	Yes
3	KRAS (G12D)	Predicted non-beneficial drugs: cetuximab, panitumumab	Yes
	TP53 (S127F)	None identified	Yes
4	CTNNB1 (S23_D32delinsN)	None identified	Yes
	KRAS (A146T)	Predicted non-beneficial drugs: cetuximab, panitumumab	Yes
5	APC (Q1291^*^)	None identified	Yes
	CDKN2A (D84N)	None identified	Yes
	MET Amplification	cabozantinib, crizotinib Predicted non-beneficial drugs: cetuximab, panitumumab	Yes
	TP53 (V143M)	None identified	Yes
6	APC (Q1291^*^)	None identified	Yes
	FGFR1 Amplification	regorafenib, lenvatinib, pazopanib, ponatinib	Yes
	MAP2K1 (K57T)	binimetinib, cobimetinib, trametinib	Yes
	TP53 (R282W)	None identified	Yes
7	ATM (Y370fs)	niraparib, olaparib, rucaparib, talazoparib	Yes
	FGFR1 Amplification	lenvatinib, pazopanib, ponatinib, regorafenib	Yes
	FGFR1/G3BP2 Fusion	lenvatinib, pazopanib, ponatinib, regorafenib	Yes

## CASE REPORTS

### Case #1

**Figure 1:**
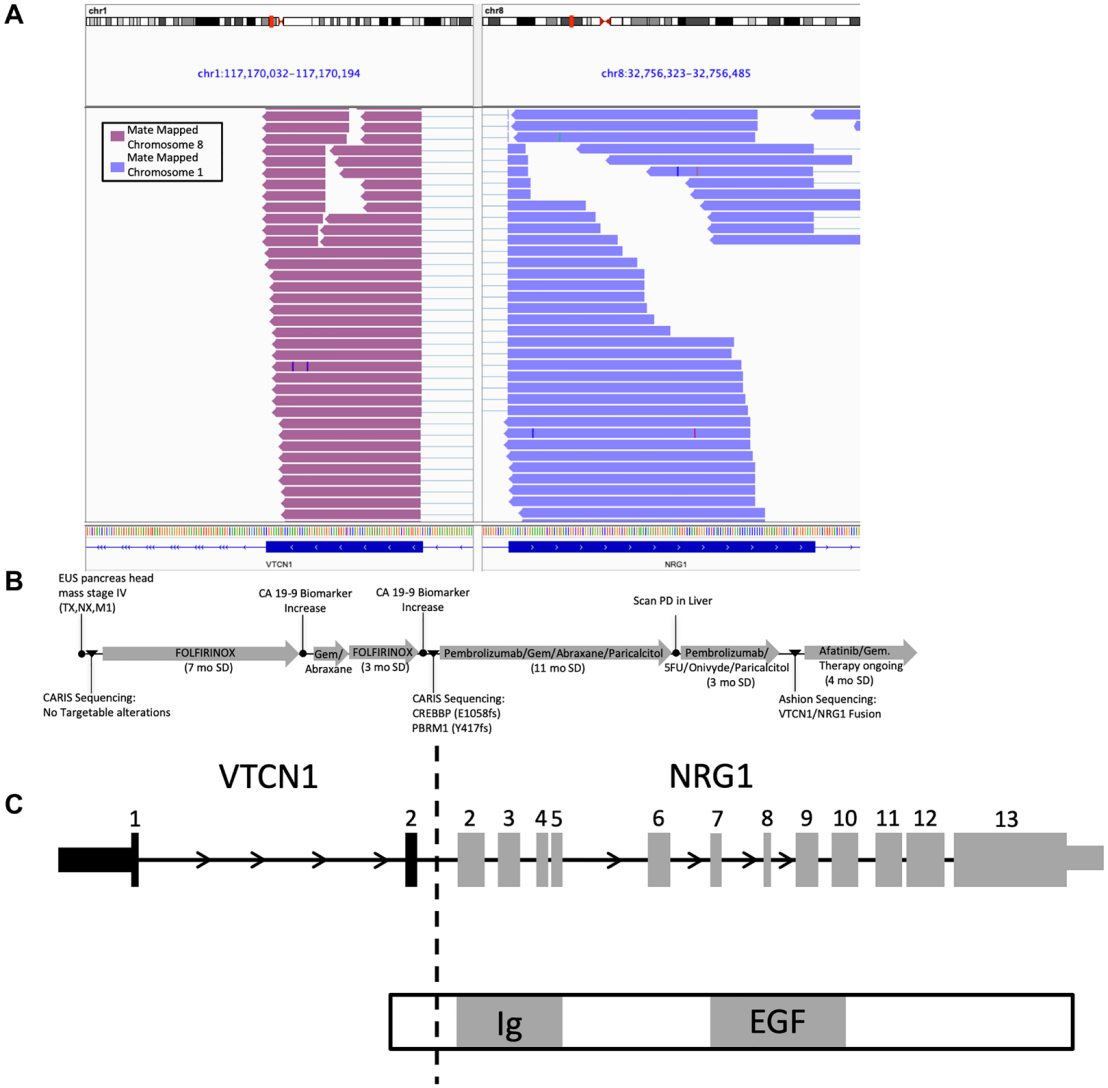
IGV graph. CASE#1 VTCN1/NRG1 fusion description and medical history of patient. (**A**) Integrated Genomics Viewer (IGV) snapshot displaying aligned supporting RNA paired end reads of the VTCN1/NRG1 fusion. (**B**) Treatment and sequencing history timeline. (**C**) Schematic drawing of the VTCN1/NRG1 fusion with expected functional domains.

A 51-year-old female was diagnosed in 2017 with malignant neoplasm of the head of the pancreas, with multiple metastatic liver lesions as well as a retroperitoneal mass. A biopsy of the retroperitoneal mass was sent for fixed genomic panel-based testing at diagnosis (Caris, Irving, TX USA). Her tumor was initially characterized as KRAS WT with several different variants of unknown significance (VUS). The genomic profile of the tumor was atypical as no KRAS mutation was detected [22]. The patient was treated with FOLFIRINOX and a year later, a liver metastasis was biopsied and sent for the same genomic panel testing. The tumor had two pathogenic mutations, CREBBP (E1058fs) and PBRM1 (Y417fs), and several other VUS. The patient was treated with several rounds of standard of Care (SOC) treatments, including chemotherapy plus a checkpoint inhibitor and the patient was stable on this treatment for one year. Upon disease progression, the patient was enrolled in this observational study and a liver metastasis was sequenced using a more comprehensive WES/RNA test, GEM ExTra^®^. A previously unidentified VTCN1/NRG1 fusion was detected which is a known driver fusion event in pancreatic cancer that lacks KRAS driver mutations [23]. Based on this finding the patient was treated with afatinib, a pan-HER tyrosine kinase inhibitor with efficacy in patients exhibiting a tumor with an NRG1 fusion [23, 24]. The patient has experienced stable disease at 4 months on this treatment at the time this report was generated. Detection of this rare actionable fusion using WES/RNA sequencing test provided new treatment opportunities for this aggressive disease with limited treatment options. (Figure 1) [25].

### Case #2

A 52-year-old female was diagnosed with GIST in 2011. She underwent surgery and was treated with imatinib until 2017. Subsequent tumor sequencing, using a panel-based test upon disease progression, demonstrated her known KIT exon 11 mutation (unknown). She was then treated in a clinical trial with ripretinib starting in 2017, followed by sunitinib in 2020. Upon disease progression, the sample was sequenced with GEM ExTra^®^ which identified CDKN2A and CDKN2B deletions, KIT (M552_K558del), KIT (N822K), KIT (Q575_Y578delinsH), KIT (Y823D). KIT (Y823D) and KIT (N822K) are two known secondary mutations associated with imatinib and sunitinib resistance and were found to be present on different alleles of KIT [26, 27]. Both secondary mutations were found within the activation loop of KIT on exon 17, demonstrating that imatinib and sunitinib may be resistant. Biallelic primary mutations have been previously characterized in GIST as having greater malignant potential, while biallelic secondary mutations have yet to be characterized [26, 27]. Our data highlights the heterogeneity of KIT secondary mutations as the main mechanism of tumor progression to KIT inhibitors in imatinib-resistant GIST patients. The GEM ExTra^®^ results suggests that multi-tyrosine kinase inhibitors including: regorafenib, ripretinib, and pazopanib, amongst others, may be best for the patient (Table 2) [28].

### Case #3

A 53-year-old female was diagnosed in 2017 with stage 4 primary neoplasm of the sigmoid colon which had metastasized to the liver. Following diagnosis, the patient was treated with FOLFIRI and bevacizumab. In 2018 she underwent sigmoidoscopy and sigmoid resection, followed by genomic panel testing (NeoGenomics, Fort Myers, FL, USA). The following was identified: FGFR1 (H810Y), PIK3CA (F934S), TP53 (S127F) none of which are targetable. To explore additional treatment opportunities, a GEM ExTra^®^ test was ordered in 2019 using a new frozen tissue sample from the original liver metastasis. The report identified KRAS (G12D), TP53 (S127F), and SMAD4 (C363Y) mutations. Presence of KRAS mutation in this sample which was not detected by previous panel testing predicted resistance to the anti-EGFR monoclonal antibodies, cetuximab and panitumumab as per NCCN guidelines.

### Case #4

A 48-year-old female was diagnosed with malignant neoplasm of the sigmoid colon in 2015. She underwent bowel resection and left adrenal biopsy in 2015. In 2016 she had laparoscopic adrenalectomy and began antineoplastic chemotherapy. From prior panel-based genomic testing she was identified as KRAS wild-type (WT) and was treated with cetuximab to which she did not respond. In 2020, GEM ExTra^®^ was performed on progression from an abdominal lesion and a rare activating KRAS (A146T) mutation was identified which leads to cetuximab resistance. Even though the exact panel used for KRAS identification is unknown, it can be inferred from the type of KRAS mutation and the allele frequency of 64%, that this mutation is most likely a truncal event which may have been missed by earlier testing, resulting in lack of response to cetuximab. Systematic review and meta-analysis of randomized, controlled trials (RCTs) evaluating anti-EGFR mAbs have reported tumors with rare RAS mutations outside of codons 12 and 13, and thus including codon 146 in genomic panels may also be predictive of resistance. The KRAS (A146T) mutation is likely an intrinsic resistance mutation which may have been detected in the original sample if exon 4 was tested [28].

### Case #5

A 67-year-old female was diagnosed with metastatic adenocarcinoma of rectal sigmoid colon in 2015. She underwent bowel resection and was treated with FOLFOX from 2015 to 2016, bevacizumab in 2016 to 2017, FOLFIRI with Ramucirumab in 2017, and FOLFIRI in the remainder of 2017. A biopsy of a new metastatic lesion from 2017 was sequenced using panel-based testing (Caris, Irving, TX, USA) and no actionable alterations were identified. The patient was treated with cetuximab + FOLFIRI based on this result and remained progression-free for about 2 years. CT scans showed progressive disease and a metastatic sample from the hip was sequenced using GEM ExTra^®^ testing which detected a high-level focal amplification of MET. While subtle increases in c-Met copy number can be detected in localized CRCs, gene amplification seems to be largely restricted to stage IV primary cancers and liver metastases [29]. MET overexpression/amplification has been associated with resistance to anti-epidermal growth factor receptor (EGFR) therapies in patients with KRAS wildtype metastatic colorectal cancer (mCRC) [30]. Thus, combining tivantinib, an inhibitor of the MET receptor tyrosine kinase, and cetuximab may be effective in patients with EGFR-resistant MET-high mCRC, [30] hence, it was determined that this patient could potentially benefit from such combination therapy.

### Case #6

A 66-year-old male was diagnosed in 2017 with metastatic colon cancer and was treated with FOLFOX and bevacizumab in 2017, and 5-FU in 2017 to 2018. He also had stereotactic radiosurgery (SRS) for the liver lesion in 2018. Subsequent treatments included FOLFIRI and bevacizumab in 2018, irinotecan and cetuximab in 2018, and regorafenib 2018 to 2019. A biopsy of the original tumor was sent for panel-based genomic testing in 2019 which identified the tumor as KRAS WT, NRAS WT, BRAF WT (Caris, Irving, TX, USA). The patient was treated with LONSURF (Trifluridine + Tipiracil) from 2019 to 2020 and was subsequently enrolled in a clinical trial. Upon disease progression, the liver metastatic lesion was sent for GEM ExTra^®^ testing in 2020 which reported a rare activating MAP2K1 (K57T) mutation, which is a known mechanism of resistance to cetuximab [31]. Based on this information, the patient was treated with standard of care treatment with FOLFOX and Avastin (bevacizumab) and has since seen decreased lung and liver lesions.

### Case #7

A 54-year-old male was diagnosed with adenocarcinoma of the pancreas and secondary neoplasm of the lung and liver in 2017. He was treated with FOLFIRINOX and had testing with FoundationOne^®^ CDx (Foundation Medicine, Cambridge, MA, USA) which identified ATM (Y370fs), BRCA1 (V1804D), and FGFR1 exon 18 non-fusion rearrangement. Additional germline testing from Invitae was performed to categorize the identified ATM and BRCA1 mutations as somatic or germline. Invitae reported no pathogenic or VUS mutations. Based on the findings of the Foundation Report, the patient was treated with a poly adenosine diphosphate-ribose polymerase (PARP) inhibitor and progressed rapidly. This germline BRCA1 (V1804D) mutation has been shown using *in-silico* and *in-vitro* models to be neutral [32, 33].

In 2019 a metastatic sample from the liver was sent for GEM ExTra^®^ testing which identified ATM (Y370fs), FGFR1 amplification, and an FGFR1/G3BP2 fusion in the metastatic tumor sample. Utilizing GEM ExTra^®^ Tumor and normal WES sequencing the aligned reads were examined to see if the BRCA1 mutation was lost via clonal evolution. Surprisingly the BRCA1 (V1804D) was detected in both the normal and tumor samples. Manual investigation revealed that the BRCA1 mutation was benign and hence was not part of the GEM ExTra^®^ report as per reporting policy. The patient most likely did not respond to PARP inhibitor due to the BRCA1 mutation being benign. These findings show paired tumor/normal analysis eliminates reporting on benign and non-pathogenic germline variants.

## DISCUSSION

NGS provides comprehensive data to inform potential targeted therapy, immunotherapy, and clinical trial options that otherwise may not be identified by other diagnostic technologies [34]. However, there are significant differences found between panel-based and comprehensive genomic profiling tests. In this study, we utilized previously developed robust comprehensive genomic platforms to interrogate the cancer genome, with the goal to determine whether the management of advanced, metastatic, refractory, or relapsed cancers can be considerably altered on the basis of the comprehensive assay design. Our assay, GEM ExTra^®^, used a combination of WES for both the tumor and matched normal tissue, and whole transcriptome sequencing for tumor profiling. We defined actionable events as reportable somatic alterations that can be targeted with the use of an existing FDA approved drug or an investigational compound in clinical trial. The list of clinically actionable genomic targets is increasing and thus facilitating patients to have more options for FDA approved therapies as well as clinical trials [35]. Recent tissue agnostic drug approvals have expanded the repertoire of available treatments which provides further evidence for the use of WES/RNA tumor-normal tests on all cancer patients with metastatic disease [36]. Therapies, such as larotrectinib and entrectinib, have been approved for patients with tumors that have NTRK gene fusions. Further, pembrolizumab has been approved for any solid tumor with a mismatch repair gene defect or a TMB greater than equal to 10 mut/Mb. These drugs were approved based on the tumor biomarker, instead of tumor origin in the body and can be picked up by comprehensive NGS profiling that captures WES and RNA seq. In this study, we report on seven unique cases of patients with advanced cancer with the goal to highlight the importance of using WES/RNA sequencing with tumor-normal subtraction for detection of fusions, rare variants, and potential acquired resistance. This is especially true for the clinical presentation of a patient suspected of having tumors, both primary and metastatic, with rare alterations or patients that present after progressing through multiple rounds of approved treatments as a means of identifying acquired resistance mutations [37].

Overall, we found that all 7 patients had at least one alteration that was previously described as a known oncogenic driver. Furthermore, we found additional actionable alterations that are known to be acquired as a secondary resistance mechanism to approved therapies. These data emphasize that tumor heterogeneity, and continual evolution of the genomic landscape contributes to disease progression. Our data highlights that molecular profiling of tumor tissue obtained from a single lesion may not always be representative of the systemic disease. Further, our data sheds light on the fact that under the pressure of targeted treatments, tumor molecular profile constantly evolves, developing resistant clones and new molecular alterations driving disease progression. Finally, our data highlights the importance of comprehensive tumor profiling and data interpretation. The 7 patients described in this paper can be grouped into two major categories: Secondary Acquired Mutations (Case #2, Case #3, Case #5, Case #6) and Missed Opportunities (Case#1, Case #4, Case #7).

### Secondary Acquired Mutations

Case #2, diagnosed as GIST, exemplifies how secondary mutations arise. Treatment of GIST with pharmacological targeting of KIT dramatically changed the clinical outcome of this disease, however resistance occurs through secondary KIT mutations that cause resistance to front line imatinib. The use of tyrosine kinase inhibitors like sunitinib and regorafenib as second- and third-line therapies, respectively, after failure on imatinib, has demonstrated limited, although significant, clinical benefit in phase III clinical trials. This is most likely due to the heterogeneity of secondary mutations in imatinib-resistant GISTs. Hence, it is crucial to sequence the metastatic lesion and understand the biology of the secondary mutations. Secondary KIT mutations are known to arise most commonly in exons 13/14 (the cytoplasmic ATP-binding domain, ABD) or exons 17/18 (the activation loop, AL), whereas primary KIT mutations predominantly affect the juxta membrane domain encoded by exon 11. Among the approved agents in GIST, sunitinib showed marked activity against KIT exon 11 mutations coupled with a secondary mutation in exon 13, whereas regorafenib was only active against KIT exon 11 mutations coupled with secondary mutations in exon 17 or exon 18. Both drugs have been shown to be active against KIT exon 11 mutations coupled with an exon 14 mutation [38]. In Case #2 both secondary mutations were found within the activation loop of KIT on exon 17 demonstrating that regorafenib, ripretinib, pazopanib, ponatinib, sorafenib, avapritinib, nilotinib or midostaurin might be the beneficial for the patient. Interestingly both resistance mutations were found on separate alleles of KIT. Biallelic primary mutations have been previous characterized in GIST as having greater malignant potential while biallelic secondary mutations have yet to be characterized [26, 27]. Our data highlights the heterogeneity of KIT secondary mutations as the main mechanism of tumor progression in response to KIT inhibitors, in imatinib-resistant GIST patients.

The importance of sequencing metastatic lesions can also be seen in cases 3, 5, and 6. All of these patients had progressed on their current treatment and NGS was employed with the hope to elucidate potentially beneficial therapies and determine secondary acquired resistance mutation. For case #3, the KRAS (G12D) mutation was identified as an alteration that predicts for lack of clinical benefit from cetuximab and thus upon identification with GEM ExTra^®^ revealed new options for the patient to participate in several different clinical trials with MEK inhibitors targeting this mutation. The use of MEK inhibitors is predicted to be beneficial in tumors with KRAS mutations [39]. However, clinical studies have suggested limited efficacy of MEK inhibitors in KRAS-mutated tumors. Hence, combinations of MEK inhibitors with other targeted therapies have been suggested to address the limited efficacy observed in these tumors [40–42]. In case #5 and case #6, two CRC patients progressed on standard of care regimens including cetuximab treatment which was based on wildtype KRAS status determined upon initial sequencing. The metastatic lesions were found to harbor MET amplification and MAP2K1 (K57T) mutation respectively. Clinical studies have identified that CRC patients who develop liver metastases harboring MPA2K1 (K57T) were insensitive to Erbitux (cetuximab) treatment. Furthermore, a combination of Vectibix (panitumumab) and Mekinist (trametinib) caused tumor regression in a colorectal cancer patient harboring MAP2K1 (K57T) [31]. Identification of the secondary resistance mutation in this case enabled optimization of next course treatment regimen with FOLFOX and Avastin which caused decreased lung and liver lesions. Similarly, MET overexpression/amplification has been associated with resistance to anti-epidermal growth factor receptor monoclonal antibody therapies in metastatic CRC [30] and clinical trials are ongoing with certain MET inhibitors in metastatic CRC patients (NCT04963283, NCT03592641).

### Missed Opportunities

Case #7 is a prime example of the importance of clinical curation in NGS analysis. This patient was sequenced at FoundationOne using tumor only and called a BRCA1 (V1804D) mutation, among others. This germline BRCA1 mutation lies within the BRCT domain 2 of BRCA1 and has been reported to be neutral using *in-silico* and *in-vitro* models [32, 33, 43]. However, there is limited data suggesting that BRCA1 (V1804D) mutation has the potential to be transcriptionally deleterious [44, 45]. The variant reporting by FoundationOne resulted in the decision to treat the patient with olaparib, and not surprisingly the patient did not respond. This highlights the need for tumor/normal pairing and importance of proper interpretation of NGS findings. The comprehensive GEM ExTra^®^ report filtered out the BRCA1 (V1804D) and instead found FGFR1 amplification and an FGFR1 fusion which may have been driving the tumor and are targetable by several FGFR inhibitors.

Case #1 shows the importance of utilizing a comprehensive NGS assay for precision oncology. The patient was sequenced several times by CARIS and failed to identify any known drivers of pancreatic cancer. Over 90% of pancreatic cancers are known to harbor activating KRAS mutations and the other 10% have oncogenic fusions, BRAF mutations, Receptor Tyrosine Kinase (RTK) mutations/amplifications, or AKT/mTOR activation [46]. Sequencing of a new metastatic lesion using GEM ExTra^®^ revealed a VTCN1/NRG1 oncogenic fusion that is most likely the driver event of the pancreatic cancer. In PDAC, pancreatic ductal adenocarcinoma, over-expression of VTCN1 has been implicated in lymph node metastasis and unfavorable prognosis [47]. The fusion partners VTCN1 and NRG1 have been previously characterized in literature but the exact translocation breakpoint is novel to this patient [48]. This driver fusion was missed earlier most likely due to the fixed panel-based testing that is designed around known fusion breakpoints. A recent study used TCGA data to identify fusions and found 4,344 recurrent fusions with 70% of those fusions defined as novel [49]. The diverse landscape of fusion biology shows that a comprehensive RNA approach like that found in GEM ExTra^®^ is more applicable to detect rare and novel fusions than fixed fusion panels. In recent studies of ERBB2 inhibitor, afatinib monotherapy resulted in rapid and partial response in PDAC patients with NRG1 fusion [23, 24]. Two other PDAC patients with NRG1 fusions also demonstrated partial remission and stable disease upon erlotinib/pertuzumab or erlotinib/trastuzumab combination therapy [50]. Finally, Case #4, the exact panel used to classify KRAS status was not able to be elucidated from the electronic medical record. Though, it is possible the previous panel-based sequencing only included the more common codon 12 and codon 13 to establish mutation status and therefore may have incorrectly classified the patient’s tumor as KRAS WT instead of as a KRAS (A146T) mutation positive. This mutation is present on exon 4 and according to NCCN Guidelines for CRC, patients with any known KRAS or NRAS mutation in exon 2, 3, 4 should not be treated with EGFR targeted monoclonal antibodies (cetuximab and panitumumab) (NCCN Guidelines, 2021). Based on current evidence, the KRAS (A146T) mutation is likely an intrinsic resistance mutation and may have been detected in the original sample if exon 4 was tested [21]. Earlier identification of this KRAS (A146T) mutation could have avoided the use of cetuximab and the associated toxicity the patient endured with no anticipated benefit. The KRAS (A146T) mutation is also potentially associated with better patient survival than KRAS WT or KRAS G12/13 mutations, and based on some published cases, the patient may have benefitted from early aggressive surgical treatment [22].

The distinguishing features compared to fixed panel tests, is that GEM ExTra^®^ employs WES and RNA seq which allows a complete picture of the molecular alterations in a single tissue sample which avoids reflex testing as well as reduces the possibility of missing an actionable alteration. In patients where all treatments have failed following serial testing, utilizing the most comprehensive test provides more chances to identify rare alterations and potentially beneficial clinical trials, which we clearly demonstrated in these case studies. The described GEM ExTra^®^ test provides physicians with a comprehensive, yet an easily interpretable report of the tumor specific genomic data along with associated FDA approved drugs and clinical trial options.

Paired tumor-normal sequencing has many advantages to tumor only sequencing by accurately reporting on true somatic mutations and identifying potential clonal hematopoietic related mutations [50]. Further, tumor only sequencing approaches have been reported to overestimate tumor mutation burden (TMB) compared with germline subtraction methods. This in turn can result in the inappropriate categorization of tumor specimens into TMB high or low and negatively affect patient outcomes to immunotherapy [51].

There are several limitations to our study. In some of the cases the tissue source for panel-based test and WES/RNA seq was not done using the same tumor sample which may have contributed to different genomic alterations found as a result of intralesional heterogeneity. Further, in a real-world setting, performing comprehensive WES and RNA seq in every single tissue sample may be confounded by availability of adequate material [52].

Our data emphasizes that tumor heterogeneity, and continual evolution of the genomic landscape contributes to disease progression. Therefore, our study joins other recently reported studies [53, 54]. that suggests including a test that interrogates tumor/normal WES as well as the transcriptome can provide a more comprehensive assessment of a patients’ tumors identifying treatment options unrecognized in somatic only panel testing. In summary, early identification of all actionable alterations in advanced cancers and upon disease progression leads to improved targeted treatment selection, patient outcomes and healthcare efficiencies.

## References

[1] El-Deiry WS, Goldberg RM, Lenz HJ, Shields AF, Gibney GT, Tan AR, Brown J, Eisenberg B, Heath EI, Phuphanich S, Kim E, Brenner AJ, Marshall JL. The current state of molecular testing in the treatment of patients with solid tumors, 2019. CA Cancer J Clin. 2019; 69:305–43. 10.3322/caac.21560. 31116423 PMC6767457

[2] Zack TI, Schumacher SE, Carter SL, Cherniack AD, Saksena G, Tabak B, Lawrence MS, Zhsng CZ, Wala J, Mermel CH, Sougnez C, Gabriel SB, Hernandez B, et al. Pan-cancer patterns of somatic copy number alteration. Nat Genet. 2013; 45:1134–40. 10.1038/ng.2760. 24071852 PMC3966983

[3] Weinstein JN, Collisson EA, Mills GB, Shaw KR, Ozenberger BA, Ellrott K, Shmulevich I, Sander C, Stuart JM, Cancer Genome Atlas Research Network. The Cancer Genome Atlas Pan-Cancer analysis project. Nat Genet. 2013; 45:1113–20. 10.1038/ng.2764. 24071849 PMC3919969

[4] Lomberk G, Dusetti N, Iovanna J, Urrutia R. Emerging epigenomic landscapes of pancreatic cancer in the era of precision medicine. Nat Commun. 2019; 10:3875. 10.1038/s41467-019-11812-7. 31462645 PMC6713756

[5] Tsimberidou AM, Iskander NG, Hong DS, Wheler JJ, Falchook GS, Fu S, Piha-Paul S, Naing A, Janku F, Luthra R, Ye Y, Wen S, Berry D, Kurzrock R. Personalized medicine in a phase I clinical trials program: the MD Anderson Cancer Center initiative. Clin Cancer Res. 2012; 18:6373–83. 10.1158/1078-0432.CCR-12-1627. 22966018 PMC4454458

[6] Radovich M, Kiel PJ, Nance SM, Niland EE, Parsley ME, Ferguson ME, Jiang G, Ammakkanavar NR, Einhorn LH, Cheng L, Nassiri M, Davidson DD, Rushing DA, et al. Clinical benefit of a precision medicine based approach for guiding treatment of refractory cancers. Oncotarget. 2016; 7:56491–500. 10.18632/oncotarget.10606. 27447854 PMC5302930

[7] Schwaederle M, Parker BA, Schwab RB, Daniels GA, Piccioni DE, Kesari S, Helsten TL, Bazhenova LA, Romero J, Fanta PT, Lippman SM, Kurzrock R. Precision Oncology: The UC San Diego Moores Cancer Center PREDICT Experience. Mol Cancer Ther. 2016; 15:743–52. 10.1158/1535-7163.MCT-15-0795. 26873727

[8] Kris MG, Johnson BE, Berry LD, Kwiatkowski DJ, Iafrate AJ, Wistuba II, Varella-Garcia M, Franklin WA, Aronson SL, Su PF, Shyr Y, Camidge DR, Sequist LV, et al. Using multiplexed assays of oncogenic rivers in lung cancers to select targeted drugs. JAMA. 2014; 311:1998–2006. 10.1001/jama.2014.3741. 24846037 PMC4163053

[9] Aisner D. Effect of expanded genomic testing in lung adenocarcinoma (LUCA) on survival benefit: The Lung Cancer Mutation Consortium II (LCMC II) experience. J Clin Oncol. 2016; 34:11510.

[10] Stockley TL, Oza AM, Berman HK, Leighl NB, Knox JJ, Shepherd FA, Chen EX, Krzyzanowska MK, Dhani N, Joshua AM, Tsao MS, Serra S, Clarke B, et al. Molecular profiling of advanced solid tumors and patient outcomes with genotype-matched clinical trials: the Princess Margaret IMPACT/COMPACT trial. Genome Med. 2016; 8:109. 10.1186/s13073-016-0364-2. 27782854 PMC5078968

[11] Bruinooge SS, Dueck AC, Gray SW, Butler NL, White CB, Smith ML. Use, attitudes, and perceptions of tumor genomic testing: Survey of TAPUR physicians. J Clin Oncol. 2019; 37:6531.

[12] Gray SW, Hicks-Courant K, Cronin A, Rollins BJ, Weeks JC. Physicians' attitudes about multiplex tumor genomic testing. J Clin Oncol. 2014; 32:1317–23. 10.1200/JCO.2013.52.4298. 24663044 PMC3992721

[13] Bryce AH, Egan JB, Borad MJ, Stewart AK, Nowakowski GS, Chanan-Khan A, Patnaik MM, Ansell SM, Banck MS, Robinson SI, Mansfield AS, Klee EW, Oliver GR, et al. Experience with precision genomics and tumor board, indicates frequent target identification, but barriers to delivery. Oncotarget. 2017; 8:27145–54. 10.18632/oncotarget.16057. 28423702 PMC5432324

[14] Ettinger DS, Wood DE, Aisner DL, Akerley W, Bauman J, Bharat A, Bruno DS, Chang JY, Chirieac LR, D’Amico TA, Dilling TJ, Dobelbower M, Gettinger S, et al. Non-small cell lung cancer, version 1. 2019; 2018. https://www.nccn.org/professionals/physician_gls/pdf/nscl.pdf. 10.6004/jnccn.2019.0059 31805526

[15] Lindeman NI, Cagle PT, Aisner DL, Arcila ME, Beasley MB, Bernicker EH, Colasacco C, Dacic S, Hirsch FR, Kerr K, Kwiatkowski DJ, Ladanyi M, Nowak JA, et al. Updated Molecular Testing Guideline for the Selection of Lung Cancer Patients for Treatment With Targeted Tyrosine Kinase Inhibitors: Guideline From the College of American Pathologists, the International Association for the Study of Lung Cancer, and the Association for Molecular Pathology. J Thorac Oncol. 2018; 13:323–58. 10.1016/j.jtho.2017.12.001. 29396253

[16] Hanna N, Johnson D, Temin S, Baker S Jr, Brahmer J, Ellis PM, Giaccone G, Hesketh PJ, Jaiyesimi I, Leighl NB, Riely GJ, Schiller JH, Schneider BJ, et al. Systemic Therapy for Stage IV Non-Small-Cell Lung Cancer: American Society of Clinical Oncology Clinical Practice Guideline Update. J Clin Oncol. 2017; 35:3484–515. 10.1200/JCO.2017.74.6065. 28806116

[17] Beitsch PD, Whitworth PW, Hughes K, Patel R, Rosen B, Compagnoni G, Baron P, Simmons R, Smith LA, Grady I, Kinney M, Coomer C, Barbosa K, et al. Underdiagnosis of Hereditary Breast Cancer: Are Genetic Testing Guidelines a Tool or an Obstacle? J Clin Oncol. 2019; 37:453–60. 10.1200/JCO.18.01631. 30526229 PMC6380523

[18] Latysheva NS, Babu MM. Discovering and understanding oncogenic gene fusions through data intensive computational approaches. Nucleic Acids Res. 2016; 44:4487–503. 10.1093/nar/gkw282. 27105842 PMC4889949

[19] Dizman N, Lyou Y, Salgia N, Bergerot PG, Hsu J, Enriquez D, Izatt T, Trent JM, Byron S, Pal S. Correlates of clinical benefit from immunotherapy and targeted therapy in metastatic renal cell carcinoma: comprehensive genomic and transcriptomic analysis. J Immunother Cancer. 2020; 8:e000953. 10.1136/jitc-2020-000953. 32661119 PMC7359179

[20] Samstein RM, Lee CH, Shoushtari AN, Hellmann MD, Shen R, Janjigian YY, Barron DA, Zehir A, Jordan EJ, Omuro A, Kaley TJ, Kendall SM, Motzer RJ, et al. Tumor mutational load predicts survival after immunotherapy across multiple cancer types. Nat Genet. 2019; 51:202–06. 10.1038/s41588-018-0312-8. 30643254 PMC6365097

[21] White T, Szelinger S, LoBello J, King A, Aldrich J, Garinger N, Halbert M, Richholt RF, Mastrian SD, Babb C, Ozols AA, Goodman LJ, Basu GD, Royce T. Analytic validation and clinical utilization of the comprehensive genomic profiling test, GEM ExTra^®^. Oncotarget. 2021; 12:726–39. 10.18632/oncotarget.27945. 33889297 PMC8057276

[22] Windon AL, Loaiza-Bonilla A, Jensen CE, Randall M, Morrissette JJD, Shroff SG. A *KRAS* wild type mutational status confers a survival advantage in pancreatic ductal adenocarcinoma. J Gastrointest Oncol. 2018; 9:1–10. 10.21037/jgo.2017.10.14. 29564165 PMC5848049

[23] Jones MR, Williamson LM, Topham JT, Lee MKC, Goytain A, Ho J, Denroche RE, Jang G, Pleasance E, Shen Y, Karasinska JM, McGhie JP, Gill S, et al. NRG1 Gene Fusions Are Recurrent, Clinically Actionable Gene Rearrangements in KRAS Wild-Type Pancreatic Ductal Adenocarcinoma. Clin Cancer Res. 2019; 25:4674–81. 10.1158/1078-0432.CCR-19-0191. 31068372

[24] Heining C, Horak P, Uhrig S, Codo PL, Klink B, Hutter B, Fröhlich M, Bonekamp D, Richter D, Steiger K, Penzel R, Endris V, Ehrenberg KR, et al. NRG1 Fusions in KRAS Wild-Type Pancreatic Cancer. Cancer Discov. 2018; 8:1087–95. 10.1158/2159-8290.CD-18-0036. 29802158

[25] Robinson JT, Thorvaldsdóttir H, Winckler W, Guttman M, Lander ES, Getz G, Mesirov JP. Integrative genomics viewer. Nat Biotechnol. 2011; 29:24–26. 10.1038/nbt.1754. 21221095 PMC3346182

[26] Lasota J, vel Dobosz AJ, Wasag B, Wozniak A, Kraszewska E, Michej W, Ptaszynski K, Rutkowski P, Sarlomo-Rikala M, Steigen SE, Schneider-Stock R, Stachura J, Chosia M, et al. Presence of homozygous KIT exon 11 mutations is strongly associated with malignant clinical behavior in gastrointestinal stromal tumors. Lab Invest. 2007; 87:1029–41. 10.1038/labinvest.3700628. 17632543

[27] Chen LL, Holden JA, Choi H, Zhu J, Wu EF, Jones KA, Ward JH, Andtbacka RH, Randall RL, Scaife CL, Hunt KK, Prieto VG, Raymond AK, et al. Evolution from heterozygous to homozygous KIT mutation in gastrointestinal stromal tumor correlates with the mechanism of mitotic nondisjunction and significant tumor progression. Mod Pathol. 2008; 21:826–36. 10.1038/modpathol.2008.46. 18488000

[28] Gausachs M, Borras E, Chang K, Gonzalez S, Azuara D, Delgado Amador A, Lopez-Doriga A, San Lucas FA, Sanjuan X, Paules MJ, Taggart MW, Davies GE, Ehli EA, et al. Mutational Heterogeneity in APC and KRAS Arises at the Crypt Level and Leads to Polyclonality in Early Colorectal Tumorigenesis. Clin Cancer Res. 2017; 23:5936–47. 10.1158/1078-0432.CCR-17-0821. 28645942 PMC5626604

[29] Zeng ZS, Weiser MR, Kuntz E, Chen CT, Khan SA, Forslund A, Nash GM, Gimbel M, Yamaguchi Y, Culliford AT 4th, D'Alessio M, Barany F, Paty PB. c-Met gene amplification is associated with advanced stage colorectal cancer and liver metastases. Cancer Lett. 2008; 265:258–69. 10.1016/j.canlet.2008.02.049. 18395971 PMC4367187

[30] Rimassa L, Bozzarelli S, Pietrantonio F, Cordio S, Lonardi S, Toppo L, Zaniboni A, Bordonaro R, Di Bartolomeo M, Tomasello G, Dadduzio V, Tronconi MC, Piombo C, et al. Phase II Study of Tivantinib and Cetuximab in Patients With KRAS Wild-type Metastatic Colorectal Cancer With Acquired Resistance to EGFR Inhibitors and Emergence of MET Overexpression: Lesson Learned for Future Trials With EGFR/MET Dual Inhibition. Clin Colorectal Cancer. 2019; 18:125–32.e2. 10.1016/j.clcc.2019.02.004. 30846365

[31] Russo M, Siravegna G, Blaszkowsky LS, Corti G, Crisafulli G, Ahronian LG, Mussolin B, Kwak EL, Buscarino M, Lazzari L, Valtorta E, Truini M, Jessop NA, et al. Tumor Heterogeneity and Lesion-Specific Response to Targeted Therapy in Colorectal Cancer. Cancer Discov. 2016; 6:147–53. 10.1158/2159-8290.CD-15-1283. 26644315 PMC4744519

[32] Lindor NM, Guidugli L, Wang X, Vallée MP, Monteiro AN, Tavtigian S, Goldgar DE, Couch FJ. A review of a multifactorial probability-based model for classification of BRCA1 and BRCA2 variants of uncertain significance (VUS). Hum Mutat. 2012; 33:8–21. 10.1002/humu.21627. 21990134 PMC3242438

[33] Easton DF, Deffenbaugh AM, Pruss D, Frye C, Wenstrup RJ, Allen-Brady K, Tavtigian SV, Monteiro AN, Iversen ES, Couch FJ, Goldgar DE. A systematic genetic assessment of 1,433 sequence variants of unknown clinical significance in the BRCA1 and BRCA2 breast cancer-predisposition genes. Am J Hum Genet. 2007; 81:873–83. 10.1086/521032. 17924331 PMC2265654

[34] McKenzie AJ, Dilks HH, Jones SF, Burris H 3rd. Should next-generation sequencing tests be performed on all cancer patients? Expert Rev Mol Diagn. 2019; 19:89–93. 10.1080/14737159.2019.1564043. 30618301

[35] Flaherty KT, Gray RJ, Chen AP, Li S, McShane LM, Patton D, Hamilton SR, Williams PM, Iafrate AJ, Sklar J, Mitchell EP, Harris LN, Takebe N, et al, and NCI-MATCH team. Molecular Landscape and Actionable Alterations in a Genomically Guided Cancer Clinical Trial: National Cancer Institute Molecular Analysis for Therapy Choice (NCI-MATCH). J Clin Oncol. 2020; 38:3883–94. 10.1200/JCO.19.03010. 33048619 PMC7676882

[36] Marcus L, Donoghue M, Aungst S, Myers CE, Helms WS, Shen G, Zhao H, Stephens O, Keegan P, Pazdur R. FDA Approval Summary: Entrectinib for the Treatment of NTRK gene Fusion Solid Tumors. Clin Cancer Res. 2021; 27:928–32. 10.1158/1078-0432.CCR-20-2771. 32967940

[37] Wang X, Zhang H, Xiaozhuo C. Drug resistance and combating drug resistance in cancer. Cancer Drug Resist. 2019; 2:141160. 10.20517/cdr.2019.10. PMC8315569 34322663

[38] Demetri GD, Reichardt P, Kang YK, Blay JY, Rutkowski P, Gelderblom H, Hohenberger P, Leahy M, von Mehren M, Joensuu H, Badalamenti G, Blackstein M, Le Cesne A, et al, and GRID study investigators. Efficacy and safety of regorafenib for advanced gastrointestinal stromal tumours after failure of imatinib and sunitinib (GRID): an international, multicentre, randomised, placebo-controlled, phase 3 trial. Lancet. 2013; 381:295–302. 10.1016/S0140-6736(12)61857-1. 23177515 PMC3819942

[39] Gilmartin AG, Bleam MR, Groy A, Moss KG, Minthorn EA, Kulkarni SG, Rominger CM, Erskine S, Fisher KE, Yang J, Zappacosta F, Annan R, Sutton D, Laquerre SG. GSK1120212 (JTP-74057) is an inhibitor of MEK activity and activation with favorable pharmacokinetic properties for sustained *in vivo* pathway inhibition. Clin Cancer Res. 2011; 17:989–1000. 10.1158/1078-0432.CCR-10-2200. 21245089

[40] Adjei AA, Cohen RB, Franklin W, Morris C, Wilson D, Molina JR, Hanson LJ, Gore L, Chow L, Leong S, Maloney L, Gordon G, Simmons H, et al. Phase I pharmacokinetic and pharmacodynamic study of the oral, small-molecule mitogen-activated protein kinase kinase 1/2 inhibitor AZD6244 (ARRY-142886) in patients with advanced cancers. J Clin Oncol. 2008; 26:2139–46. 10.1200/JCO.2007.14.4956. 18390968 PMC2718422

[41] Infante JR, Somer BG, Park JO, Li CP, Scheulen ME, Kasubhai SM, Oh DY, Liu Y, Redhu S, Steplewski K, Le N. A randomised, double-blind, placebo-controlled trial of trametinib, an oral MEK inhibitor, in combination with gemcitabine for patients with untreated metastatic adenocarcinoma of the pancreas. Eur J Cancer. 2014; 50:2072–81. 10.1016/j.ejca.2014.04.024. 24915778

[42] Hochster HS, Uboha N, Messersmith W, Gold PJ, ONeil BH, Cohen D, Denlinger C, Cohen S, Leichman CG, Leichman L, Lenz HJ. Phase II study of selumetinib (AZD6244, ARRY-142886) plus irinotecan as second-line therapy in patients with K-RAS mutated colorectal cancer. Cancer Chemother Pharmacol. 2015; 75:17–23. 10.1007/s00280-014-2609-3. 25322874

[43] Thouvenot P, Ben Yamin B, Fourrière L, Lescure A, Boudier T, Del Nery E, Chauchereau A, Goldgar DE, Houdayer C, Stoppa-Lyonnet D, Nicolas A, Millot GA. Functional Assessment of Genetic Variants with Outcomes Adapted to Clinical Decision-Making. PLoS Genet. 2016; 12:e1006096. 10.1371/journal.pgen.1006096. 27272900 PMC4894565

[44] Ostrow KL, McGuire V, Whittemore AS, DiCioccio RA. The effects of BRCA1 missense variants V1804D and M1628T on transcriptional activity. Cancer Genet Cytogenet. 2004; 153:177–80. 10.1016/j.cancergencyto.2004.01.020. 15350310

[45] Chang S, Biswas K, Martin BK, Stauffer S, Sharan SK. Expression of human BRCA1 variants in mouse ES cells allows functional analysis of BRCA1 mutations. J Clin Invest. 2009; 119:3160–71. 10.1172/JCI39836. 19770520 PMC2752086

[46] Aguirre AJ. Oncogenic NRG1 Fusions: A New Hope for Targeted Therapy in Pancreatic Cancer. Clin Cancer Res. 2019; 25:4589–91. 10.1158/1078-0432.CCR-19-1280. 31164372 PMC6679796

[47] Chen X, Tao L, Yuan C, Xiu D. The prognostic value of B7-H4 in pancreatic cancer: Systematic review and meta-analysis. Medicine (Baltimore). 2018; 97:e0088. 10.1097/MD.0000000000010088. 29561406 PMC5895337

[48] Jonna S, Feldman RA, Swensen J, Gatalica Z, Korn WM, Borghaei H, Ma PC, Nieva JJ, Spira AI, Vanderwalde AM, Wozniak AJ, Kim ES, Liu SV. Detection of NRG1 Gene Fusions in Solid Tumors. Clin Cancer Res. 2019; 25:4966–72. 10.1158/1078-0432.CCR-19-0160. 30988082 PMC7470623

[49] Vellichirammal NN, Albahrani A, Banwait JK, Mishra NK, Li Y, Roychoudhury S, Kling MJ, Mirza S, Bhakat KK, Band V, Joshi SS, Guda C. Pan-Cancer Analysis Reveals the Diverse Landscape of Novel Sense and Antisense Fusion Transcripts. Mol Ther Nucleic Acids. 2020; 19:1379–98. 10.1016/j.omtn.2020.01.023. 32160708 PMC7044684

[50] Mandelker D, Ceyhan-Birsoy O. Evolving Significance of Tumor-Normal Sequencing in Cancer Care. Trends Cancer. 2020; 6:31–39. 10.1016/j.trecan.2019.11.006. 31952779 PMC8923150

[51] Parikh K, Huether R, White K, Hoskinson D, Beaubier N, Dong H, Adjei AA, Mansfield AS. Tumor Mutational Burden From Tumor-Only Sequencing Compared With Germline Subtraction From Paired Tumor and Normal Specimens. JAMA Netw Open. 2020; 3:e200202. 10.1001/jamanetworkopen.2020.0202. 32108894 PMC7049088

[52] Benayed R, Offin M, Mullaney K, Sukhadia P, Rios K, Desmeules P, Ptashkin R, Won H, Chang J, Halpenny D, Schram AM, Rudin CM, Hyman DM, et al. High Yield of RNA Sequencing for Targetable Kinase Fusions in Lung Adenocarcinomas with No Mitogenic Driver Alteration Detected by DNA Sequencing and Low Tumor Mutation Burden. Clin Cancer Res. 2019; 25:4712–22. 10.1158/1078-0432.CCR-19-0225. 31028088 PMC6679790

[53] LoRusso PM, Sekulic A, Sosman JA, Liang WS, Carpten J, Craig DW, Solit DB, Bryce AH, Kiefer JA, Aldrich J, Nasser S, Halperin R, Byron SA, et al. Identifying treatment options for BRAFV600 wild-type metastatic melanoma: A SU2C/MRA genomics-enabled clinical trial. PLoS One. 2021; 16:e0248097. 10.1371/journal.pone.0248097. 33826614 PMC8026051

[54] Rusch M, Nakitandwe J, Shurtleff S, Newman S, Zhang Z, Edmonson MN, Parker M, Jiao Y, Ma X, Liu Y, Gu J, Walsh MF, Becksfort J, et al. Clinical cancer genomic profiling by three-platform sequencing of whole genome, whole exome and transcriptome. Nat Commun. 2018; 9:3962. 10.1038/s41467-018-06485-7. 30262806 PMC6160438

