# Addendum: The value of comprehensive genomic sequencing to maximize the identification of clinically actionable alterations in advanced cancer patients: a case series

**DOI:** 10.18632/oncotarget.28294

**Published:** 2022-10-20

**Authors:** Kevin Drenner, Gargi D. Basu, Laurie J. Goodman, Audrey A. Ozols, Janine R. LoBello, Thomas Royce, Michael S. Gordon, Erkut H. Borazanci, Margaux A. Steinbach, Jeffrey Trent, Sunil Sharma

**Affiliations:** ^1^Translational Genomic Research Institute (Tgen), Phoenix, AZ 85004, USA; ^2^Ashion Analytics, LLC, Phoenix, AZ 85004, USA; ^3^HonorHealth Research Institute, Scottsdale, AZ 85258, USA; ^*^These authors contributed equally to this work

**Consent was added to this article:** The study, protocol # HRI-0029 is an IRB approved clinical study, WIRB # 20182804, and all patients signed informed consent to participate.

Original article: Oncotarget. 2021; 12:1836–1847. 1836-1847. https://doi.org/10.18632/oncotarget.28046

